# Oral health status in relation to socioeconomic and behavioral factors among pregnant women: a community-based cross-sectional study

**DOI:** 10.1186/s12903-019-0801-x

**Published:** 2019-06-17

**Authors:** Marzie Deghatipour, Zahra Ghorbani, Shahla Ghanbari, Shahnam Arshi, Farnaz Ehdayivand, Mahshid Namdari, Mina Pakkhesal

**Affiliations:** 1 Dental Research Center, Research Institute of Dental Sciences, Dental School, Shahid Beheshti University of Medical Sciences, Tehran, Iran; 2 Community Oral Health Department, School of Dentistry, Shahid Beheshti University of Medical Sciences, Daneshjoo Blvd, Tehran, IR 19834 Iran; 3 Department of Health Education and Health Promotion, Deputy for Health affairs, Shahid Beheshti University of Medical sciences, Tehran, Iran; 4 Deputy for Health affairs, Shahid Beheshti University of Medical Sciences, Tehran, Iran; 5 Obstetrician and gynecologist, Deputy of Health Affairs, Shahid Beheshti University of Medical Sciences, Tehran, Iran; 6 Of Biostatistics, Department Of Community Oral Health, School Of Dentistry, Shahid Beheshti University Of Medical Sciences, Tehran, Iran; 7 Community Oral Health Department, School of Dentistry, Golestan University of Medical Sciences, Gorgan, Iran

**Keywords:** Oral health, Pregnant women, Dental care behaviors

## Abstract

**Background:**

Oral health of women during pregnancy is an important issue. Not only it can compromise pregnancy outcomes, but also it may affect their newborn’s overall health. The aim of this study was to assess the oral health status and associated factors in pregnant women.

**Methods:**

A cross-sectional study was conducted amongst 407 pregnant women in the second and third trimester of pregnancy in Varamin, Iran. Oral health status was examined, and demographic, socioeconomic status and dental care behavior data were collected. Oral health indices included periodontal pocket, bleeding on probing (BOP) and decayed, missed, filled teeth (DMFT). Regression analysis of DMFT was used to study the association between demographic, dental care behaviors indicators and outcome variables using the count ratios (CR) and 95% confidence intervals (CI).

**Results:**

The mean (SD, Standard Deviation) age of participants was 27.35 (5.57). Daily brushing, flossing habit were observed in 64.1, and 20.6% of mothers, respectively. Mean (SD) of DMFT, D, M, F were 10.34(5.10), 6.94(4.40), 2.22 (2.68) and 1.19(2.23), respectively. Women older than 35 years had significantly more DMFT [CR = 1.35 (95% CI 1.13; 1.60)], less D [CR = 0.75 (95% CI 0.59; 0.94)], and more M [CR = 3.63 (95% CI 2.57; 5.14)] compared to women under 25 years after controlling for education and dental care behaviors. Women with academic education had significantly less decayed teeth [CR = 0.63 (95% CI 0.48; 0.84)], compared to women with under 12 years of education.

**Conclusions:**

Oral health status of pregnant women was not satisfactory, having an average of seven decayed teeth in their mouth.

## Background

Pregnancy is a natural process accompanied with considerable physiological and hormonal changes in women’s body, including oral cavity [1]. There are many common oral problems in pregnancy such as pregnancy gingivitis, benign gingival lesions, tooth mobility, tooth erosion, dental caries, and periodontitis [2]. Oral health is an important issue to general health of both the expectant woman and her infant [3]. Evidence showed that insufficient of oral health care during pregnancy can have negative outcomes for both mothers and their newborns [4].

According to a systematic review, the relationship between pregnancy and gingivitis was confirmed. The characteristics of pregnancy-associated gingivitis are similar to common plaque-related gingivitis but with more severity; the severity being correlated with blood steroid hormone levels [3]. Periodontal disease during pregnancy has been criticized to be associated with adverse perinatal outcomes, including preeclampsia, preterm delivery, low birth weight, increased fetal death and newborn’s care time in neonatal care unit [5–7] Therefore, women should be given proper oral hygiene and oral health preventive services before, during and even after child birth [8].

Mother’s oral health behavior during pregnancy, such as dental visits, oral hygiene, and consumption of sweets have a significant effect on their oral health during pregnancy and on their children’s oral health in the future [9, 10]. Expectant women should be counseled to perform routine brushing and flossing, to avoid consuming excessive amounts of sugary snacks and drinks, and to consult a dentist during pregnancy [11].

Pregnant women may not be aware of the effects of their oral health on the fetus and their pregnancy outcomes [12]. Plenty of studies have shown pregnant women had negative attitude towards their oral health care and dental care utilization in pregnancy period [13, 14]. Patients and dentists usually avoid dental treatment during pregnancy because of absence of clinical guidelines for dental management in pregnancy, lack of practice standards, and anxiety about fetal safety during dental procedures [15].

Although oral health in pregnancy is an important issue in pregnancy health, few epidemiological studies have reported clinical oral health indices in the population. A search in PubMed database with Mesh keywords of “pregnant women” and “oral health” in May 2019 resulted in finding 34 papers, from which only four contained information regarding clinical oral health examination [16–19]. This shortage of literature may be because of neglected oral health as a part of needed maternal care and less dental visits in pregnancy.

Oral health of Iranian adults (aged 35–44) has been studied in a recent epidemiological study in 2012 [20]; however, it did not include pregnant women.

The aim of this study was to get baseline data about oral health status and dental care behaviors of pregnant women in Varamin, a partially deprived region in the southern part of Tehran Province, Iran.

This information may be helpful to design and plan interventions regarding oral health promotion among this target population.

## Methods

This study was approved by the Committee of Ethics in Research Affairs of Dental School, Shahid Beheshti University of Medical sciences. After explanation of the study objectives, an informed consent was obtained for the participation. Women filled the consent form, and signed it for participation. There was a participant under 16 years whose consent form was signed by her father as her legal guardian according the local rules.

This community-based cross-sectional study was performed to provide the baseline data for a community oral health promoting intervention to be implemented in “Pishva”;“Pakdasht”, both regions located in Varamin, a southern part of Tehran Province in Iran. At the 2016 census, Pishva’s population was 86,601 and Pakdasht was 350,966 (https://www.amar.org.ir/english/Population-and-Housing-Censuses). Pishva and Pakdasht are similar regions in terms of socioeconomic status. Data gathering started in July 2016, and lasted for eight months.

### Sample size and subject recruitment

In this study in order to estimate the mean Decayed, Missed, Filled teeth (DMFT) in pregnant women, according to the previous study on decay-missing-filled (DMF) of Iranian pregnant women [21], the Standard Deviation (SD), was found to be 3, considering 95% confidence with an error equal to 0.3 a sample of approximately 387 subjects was needed to be participated in the study.

The target population was pregnant women in the second/third trimester of pregnancy living in Varamin. Women with known systemic disease, with high-risk pregnancy, age under 15 years, and on long-term medication were excluded. Recruitment was carried out in all the 17 health care centers in Pishva (7 health care centers) and Pakdasht (10 health care centers), where more than 70% of pregnant women residing in those regions usually receive their maternal care from these public health centers. All of the health care centers provided free maternal and child care for their target groups at the same conditions. All 407 pregnant women in their second/third trimester of pregnancy who received prenatal care from the health centers were recalled toparticipate in the study.

### Study design

Two dentists were trained according to the World Health Organization (WHO) oral health surveys basic methods (15), and were calibrated clinically using 10 patients in a two-day calibration workshop. The intra- and inter-examiner reliability of measuring dental caries and pocket depth were investigated for both examiners. The mean inter-examiner agreement obtained in this activity was Kappa = 0.85. Dental and periodontal examinations were taken in in maternity care room of Pishva and Pakdasht health centers on an ordinary seat.. Teeth were dried by cotton rolls, and oral examination was performed using battery-operated lights, mouth mirror, and community periodontal index probe according to the recommendations of WHO [22].

For assessing periodontal status, BOP and periodontal pocket were detected. Gingivae of all teeth present in participants’ mouth examined by inserting the tip of the WHO CPI probe between the gingiva and the tooth to assess absence or presence of bleeding response. All participant’s teeth present in the mouth were examined for absence or presence of gingival.

bleeding and absence or presence of periodontal pockets; pocket depth is measured with the WHO CPI periodontal probe. The probe tip inserted gently into six sites of the gingival sulcus or pocket and the full extent of the sulcus or pocket explored.

After oral examination and interview, all the study participants were given a tube of fluoridated tooth paste and a tooth brush. The brushing was demonstrated to all and were advised to brush twice a day.

### Variables

Outcome variables collected through oral examination included DMFT, bleeding on probing at least one site, and having periodontal pocket > 3.5 mm in at least one tooth.

Explanatory variables were collected via face to face interviews using a structured standard questionnaire. The questionnaire included information regarding the explanatory variables including pregnant women’s age, trimester of pregnancy (second/third), educational background, job, frequency of tooth brushing/ dental flossing/ sweets consumption, dental visit, and cause of last dental visit.

Women’s education was asked with 5 possible answers from “illiterate” to “university degrees” which was then classified to “Less than 12 years”, “12 years”, and “More than12 years” for further analysis. The responses for brushing/flossing/sweet consumption were dichotomized into “once a day or more” or “less than once a day”. Dental visit information was gathered through a yes/no question: “Have you had any dental visits in the previous 12 months?” the cause of the last dental visit was asked and dichotomized into treatment and checkup (consultation, checkup). Mother’s job was asked with 2 possible answers from “house keeper” to “employed”.

### Statistical analysis

All the data were entered in a data entry form and statistical analysis was performed using SPSS version 21 and STATA/SE 11. For bivariate statistical analysis, Independent Sample T-test, Chi-square, one-way ANOVA and Mann-Whitney U tests were used. For modeling DMFT, D, and M, negative binomial models were used, and for BOP and periodontal pocket, binary logistic regressions were applied. Because of plenty of zeros in F, zero-inflated negative binomial model was used. Unadjusted and adjusted models were used to study the association between explanatory variables and outcome variables, considering *P*-value less than 0.05 as statistically significant level. Model 1 included age group. Model 2 consisted of age group, and trimester of pregnancy. Education was added to Model 3 while oral health behaviors (brushing habit, flossing habit, dental visit and sweet consumption) were added to Model 4.

## Results

**Fig. 1:**
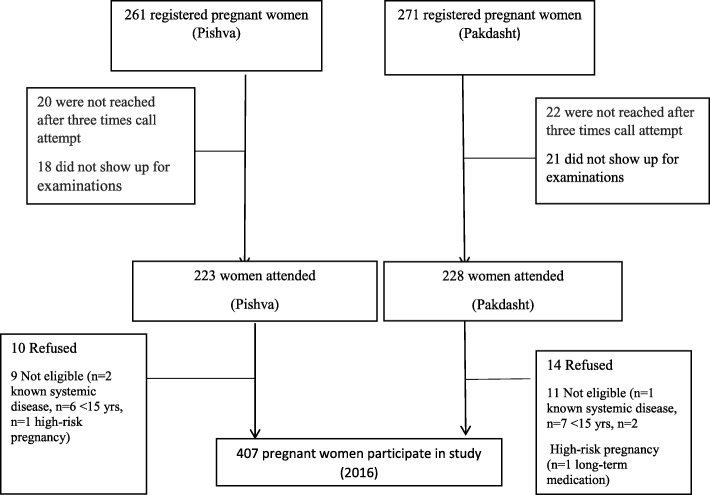
Flowchart of study design

From the 532 registered women, 451 attended the examination session. Also, 38 pregnant women (in Pishva) and 43 pregnant mothers (in Pakdasht) did not attend the examination exactly. The flowchart of study design is provided in Fig. 1. The mean age (SD) of the 407 pregnant women was 27.35(5.57). Approximately, half of the participants were 25–35 years old, ranging between 15 to 44 years. A majority of the expectant women (59.7%) were in second trimester of pregnancy. Approximately, half of the women were educated less than 12 years. Only 1.7% of women were employed. The frequency of daily brushing among participated women was 64.1% and One out of four women had daily flossing habit (Table 1).

**Table 1:** Demographic, socioeconomic and oral health of pregnant women (*n* = 407)

		Variables	N (%)	DMF Mean (SD)	D Mean (SD)	M Mean (SD)	F Mean (SD)	Having periodontal pocket > 3.5 mm N(%)	Having bleeding on probing N(%)
Demographic variables	Age group	15–25	162 (39.8)	9.22 (5.29)	7.21 (4.66)	1.30 (1.90)	0.71 (1.63)	27 (16.7%)	128 (79.1%)
		25–35	204 (50.1)	10.82 (4.81)	7.00 (4.30)	2.47 (2.64)	1.35 (2.37)	30 (14.7%)	152 (74.1%)
		35–44	41 (10.1)	12.39 (4.84)	5.54 (3.58)	4.59 (3.67)	2.27 (3.04)	3 (7.3%)	27 (65.9%
		*P-value*		**< 0.001**	0.09	**< 0.001**	**< 0.001**	0.32	0.06
	Trimester of pregnancy	Second	243 (59.7)	10.17 (5.11)	6.83 (4.43)	2.15 (2.45)	1.19 (2.21)	27 (11.1%)	177 (72.8%)
		Third	164 (40.3)	10.59 (5.10)	7.09 (4.36)	2.32 (2.99)	1.18 (2.28)	33 (20.1%)	130 (79.3%)
		*P-value*		0.43	0.62	0.42	0.71	**< 0.05**	0.13
Indicators of socioeconomic status	Job	House keeper	400 (98.3)	10.31 (5.11)	6.93 (4.40)	2.20 (2.68)	1.19 (2.24)	59 (14.8%)	302 (75.5%)
		employed	7 (1.7)	12.00 (4.47)	7.43 (4.46)	3.43 (2.76)	1.14 (1.77)	1 (14.3%)	5 (71.4%)
		*P-value*		0.57	0.74	0.69	0.45	0.97	0.80
	Education (years of schooling)	Less than 12 years	224 (55)	10.41 (5.47)	7.40 (4.69)	2.22 (2.69)	0.79 (1.84)	33 (14.7%)	171 (76.3%)
		12 years	156 (38.3)	10.38 (4.75)	6.60 (4.00)	2.23 (2.74)	1.56 (2.49)	25 (16.0%)	116 (74.4%)
		More than12 years	27 (6.6)	9.48 (3.85)	5.04 (3.44)	2.07 (2.25)	2.37 (2.91)	2 (7.4%)	20 (74.1%)
		*P-value*		0.66	**< 0.05**	0.96	**< 0.001**	0.65	0.22
Dental care behaviors	Brushing Habit	Once a day or more	261 (64.1)	10.52 (4.73)	7.24 (4.39)	2.16 (2.39)	1.12 (2.01)	40 (15.3%)	148 (56.7%)
		Less than once a day	146 (35.9)	10.02 (5.70)	6.40 (4.38)	2.32 (3.14)	1.30 (2.59)	20 (13.7%)	79 (54.1%)
		*P-value*		**< 0.05**	0.35	**< 0.05**	**< 0.05**	0.65	0.21
	Flossing Habit	once a day or more	84 (20.6)	9.93 (4.92)	7.21 (4.27)	1.77 (2.17)	0.94 (2.23)	6 (7.1%)	49 (58.3%)
		Less than once a day	323 (79.4)	10.45 (5.15(	6.86 (4.44)	2.33 (2.79)	1.25 (2.23)	54 (16.7%)	185 (57.3%)
		*P-value*		0.70	0.88	0.19	0.39	**< 0.05**	**< 0.01**
	Dental Visit	Yes	205 (50.4)	11.09 (5.35)	6.99 (4.67)	2.56 (3.02)	1.54 (2.51)	27 (13.2%)	120 (58.5%)
		No	202 (49.6)	9.58 (4.73)	6.88 (4.12)	1.87 (2.24)	0.83 (1.85)	33 (16.3%)	97 (48.0%)
		*P-value*		0.42	0.29	**< 0.01**	**< 0.001**	0.36	0.98
	Sweet Consumption	once a day	274 (67.3)	10.28 (5.09)	6.75 (4.35)	2.15 (2.63)	1.38 (2.50)	37 (13.5%)	144 (52.5%)
		more than once a day	133 (32.7)	10.46 (5.13)	7.32 (4.49)	2.35 (2.78)	0.79 (1.47)	23 (17.3%)	73 (54.8%)
		*P-value*		0.78	0.71	0.74	**< 0.001**	0.31	0.07
	Cause of last visit	checkup	86 (21.1)	8.26 (5.15)	5.92 (4.33)	1.02 (1.82)	1.31 (2.66)	16 (18.6%)	34 (59.6%)
		treatment	321 (78.9)	10.90 (4.95)	7.21 (4.38)	2.54 (2.78)	1.15 (2.11)	44 (13.7%)	162 (70.1%)
		*P-value*		0.33	0.92	**< 0.01**	0.07	0.25	0.17
All			407 (100%)	10.34 (5.10)	6.94 (4.40)	2.22 (2.68)	1.19 (2.23)	113 (27%)	307 (75%)

For comparison between D, M, F, DMFT and demographic, socioeconomic, oral health determinants Independent T-test was used

For comparison between D, M, F, DMFT and age and educational background groups One-way ANOVA test was used

For comparison between Periodontal Pocket > 3 mm, Bleeding and demographic, socio economic, oral health determinants Chi-square or Fisher exact test was used

Bold: relationship significant at the 5% level

The mean (SD) DMFT, D, M and F in our study participant group were 10.34(5.10), 6.94(4.40), 2.22 (2.68) and 1.19(2.23), respectively. Dental decay accounted for approximately 67% of DMFT while dental filling accounted for only 10%.

The percentage of D/DMFT in 15–25 years age group was 78% and in 35–44 years age was 44%, while percentage of M/DMFT in 15–25 years and 35–44 years age group were 14 and 44%, respectively. The contribution of filling teeth among DMFT was low in all age groups. Women in third trimester of pregnancy had significantly more periodontal pocket > 3.5 mm, compared to women in second trimester (*p* < 0.05).

Approximately three quarters of participants had BOP while 27% had periodontal pocket > 3.5 mm. Among those having periodontal pocket, the mean (SD) of total number of teeth with periodontal pocket > 3.5 mm was 3.9 (4.47).

As reported in Table 1, less dental caries and more dental fillings were observed among pregnant women with more than 12 years education (*p* < 0.01). Bleeding on probing and having periodontal pocket more than 3.5 mm were significantly more in women using dental floss less than once a day (p < 0.05). Expectant mothers who had dental visit in the previous years had significantly more filled and missed teeth (p < 0.01). More missing teeth were observed in participants who had visited a dentist to get a treatment (p < 0.01).

Women older than 35 years had 1.3 times more DMFT [CR = 1.35 (95% CI 1.13; 1.60)] (Table 2), 0.25 times less decayed teeth [CR = 0.75 (95% CI 0.59; 0.94)] (Table 3), and 3.5 times more missing teeth [CR = 3.63 (95% CI 2.57; 5.14)] compared to women younger than 25 (Table 4)**.**

**Table 2:** Association between demographic and dental care behaviors with number of DMFT

Variables		Model 1	Model 2	Model 3	Model 4
		CR(95%CI)	CR(95%CI)	CR(95%CI)	CR(95%CI)
Age	15–25 25–35 35–45	1 **1.15(1.03;1.29)** **1.32(1.12;1.1.59)**	1 **1.17 (1.05;1.30)** **1.34(1.13;1.60)**	1 **1.17(1.05;1.31)** **1.34(1.13;1.60)**	1 **1.18(1.06;1.31)** **1.35 (1.13;1.60)**
Trimester	Second Third		1 1.04(0.94;1.16)	1 1.04(0.94;1.15)	1 1.04 (0.94;1.15)
Education	Less than 12 years 12 years More than 12 years			1 0.99(0.89;1.10) 0.92(0.74;1.13)	1 0.97(0.87;1.08) 0.89(0.72;1.10)
Brushing Habit	once a day Less than once a day				1 1.04(0.94;1.16)
Flossing Habit	Less than once Once a day or more				1 0.95 (0.84;1.08)
Dental Visit	Yes No				1 **0.86(0.77;0.95)**
Sweet Consumption	Once More than once				1 1.02(0.92;1.14)

Negative binomial regression

Bold: relationship significant at the 5% level

Model 1: adjusted for age group

Model 2: adjusted for age group, trimester of pregnancy

Model 3: adjusted for age group, trimester of pregnancy, education

Model 4 adjusted for age group, trimester of pregnancy, education, oral health behaviors

**Table 3:** Association between demographic and dental care behaviors with number of decayed teeth

Variables		Model 1 CR (95%CI)	Model 2 CR (95%CI)	Model 3 CR (95%CI)	Model 4 CR (95%CI)
Age	15–25 25–35 35–45	1 0.97(0.84;1.11) **0.76(0.60;0.97)**	1 0.97(0.85;1.11) **0.77(0.60;0.98)**	1 0.98(0.86;1.13) **0.75(0.59;0.94)**	1 0.98(0.86;1.13) **0.75(0.59;0.94)**
Trimester	Second Third		1 1.03(0.90;1.18)	1 1.01(0.88;1.15)	1 1.00(0.88;1.14)
Education	Less than 12 years 12 years More than 12 years			1 0.87(0.76;1.00) **0.66(0.50;0.88)**	1 **0.86(0.75;0.99)** **0.63(0.48;0.84)**
Brushing habit	once a day Less than once a day				1 **1.15 (1.00;1.31)**
Flossing habit	Less than once Once a day or more				1 1.10(0.94;1.29)
Dental visit	Yes No				1 0.96(0.84;1.09)
Sweet Consumption	Once More than once				1 1.05(0.92;1.21)

Negative binomial regression

CR, count ratio; CI, 95% confidence interval

Bold: relationship significant at the 5% level

Model 1: adjusted for age group

Model 2: adjusted for age group, trimester of pregnancy

Model 3: adjusted for age group, trimester of pregnancy, education

Model 4 adjusted for age group, trimester of pregnancy, education, oral health behaviors

**Table 4:** Association between demographic and dental care behaviors with number of missing teeth

Variables	Levels	Model 1 CR(95%CI)	Model 2 CR(95%CI)	Model 3 CR(95%CI)	Model 4 CR(95%CI)
Age	15–25 25–35 35–45	1 **1.90(1.50;2.41)** **3.53(2.49;5.02)**	1 **1.90(1.50;2.41)** **3.59(2.53;5.10)**	1 **1.90(1.49;2.41)** **3.59(2.53;5.11)**	1 **1.96(1.55;2.49)** **3.63(2.57;5.14)**
Trimester	Second Third		1 1.13(0.91;1.41)	1 1.13(0.91;1.41)	1 1.10(0.89;1.37)
Education	Less than 12 years 12 years More than 12 years			1 1.01(0.80;1.26) 1.02(0.65;1.59)	1 0.98(0.79;1.23) 1.02(0.65;1.59)
Brushing Habit	Once a day Less than Once a day				1 0.90(0.72;1.12)
Flossing Habit	Less than Once a day Once a day or more				1 **0.75(0.57;0.99)**
Dental Visit	Yes No				1 **0.75(0.61;0.93)**
Sweet Consumption	Once More than once				1.15(0.92;1.44)

Negative binomial regression

CR, count ratio; CI, 95% confidence interval

Bold: relationship significant at the 5% level

Model 1: adjusted for age group

Model 2: adjusted for age group, trimester of pregnancy

Model 3: adjusted for age group, trimester of pregnancy, education

Model 4 adjusted for age group, trimester of pregnancy, education, oral health behaviors

Also, women who haven’t visited a dentist in the previous years had significantly less DMFT [CR = 0.86 (95% CI 0.77; 0.95)]. Having more than 12 years education was associated with less dental caries, even after controlling for dental care behaviors [CR = 0.63 (95% CI 0.48; 0.84)]. Also, women who had brushed their teeth less than once a day had significantly more decayed teeth [CR = 1.15 (95% CI 1.00; 1.31)].

Additionally, women who haven’t visited a dentist in the previous year had significantly less dental missing [CR = 0.75(95% CI 0.61; 0.93)].

Tables 5 illustrates that filling teeth increased by age but after controlling for dental care behaviors, this association was not significant any more.

**Table 5:** Association between demographic and dental care behaviors with number of filling teeth

Variables	Levels	Model 1 CR(95%CI)	Model 2 CR(95%CI)	Model 3 CR(95%CI)	Model 4 CR(95%CI)
Age	15–25 25–35 35–45	1 1.40(0.89;2.21) **2.12(1.14;3.92)**	1 1.35(0.84;2.15) **2.09 (1.13;3.88)**	1 1.13(0.71;1.79) **2.03(1.09;3.79)**	1 1.06(0.62;1.81) 1.69(0.92;3.12)
Trimester	Second Third		1 1.14(0.75;1.72)	1 1.26(0.82;1.93)	1 1.02(0.65;1.61)
Education	Less than 12 years 12 years More than 12 years			1 1.25(0.81;1.92) 1.44(0.77;2.70)	1 1.27(0.82;1.97) 1.43(0.79;2.59)
Brushing Habit	Once a day Less than Once a day				1 0.75(0.47;1.19)
Flossing Habit	Less than Once a day Once a day or more				1 1.34(0.78;2.32)
Dental Visit	Yes No				1 0.81(0.54;1.22)
Sweet Consumption	Once More than once				1 **0.60(0.37;0.99)**

Zero inflated negative binomial regression

CR, count ratio; CI, 95% confidence interval

Bold: relationship significant at the 5% level

Model 1: adjusted for age group

Model 2: adjusted for age group, trimester of pregnancy

Model 3: adjusted for age group, trimester of pregnancy, education

Model 4 adjusted for age group, trimester of pregnancy, education, dental care behaviors

Regression analysis for BOP reveal that sweet consumption more than once a day and flossing less than once a day were positively related to BOP. The detailed table is provided in the appendix 1.

## Discussion

The finding of the present study showed the high prevalence of gingival diseases and dental caries in pregnant women. Having a mean of seven decayed teeth in mother’s mouth, can be a prelude to dental caries in newborns’ mouth via bacterial transmission. The mean DMFT was 10.34 which was higher than the mean DMFT of pregnant women in Arak and Shiraz, two major cities in Iran [21, 23]. Differences in the DMFT in different areas of Iran may be due to the differences in socioeconomic status of the studied regions. The Iranian National Oral Health Survey-2012 did not include pregnant women. According the results of this study, the mean DMFT in 35–44 years old females was 13.07 [20], being similar to this age group DMFT in the present study.

Dental decay accounted for 67% of DMFT while dental filling accounted for only 10 % of DMFT, showing that most of dental caries have been left untreated. In older age groups, the percentage of D was lower, and the percentage of missing teeth were higher than the younger age groups. It indicates inappropriate use of dental services, leading to extraction of decayed teeth rather to reserve them by dental treatment. The private-dominant dental care system, financial barriers, and insufficient accessibility of dental care may be the underlying causes [24, 25].

This high proportion of D to DMFT is also in agreement with studies on pregnant women in developing countries like India [26], and rural Sri Lanka [17]. But it was in contrast with a study from Australia [27], where F was the largest portion of DMFT. This may be because of the differences in socioeconomic status, oral health beliefs, access to dental care and patterns of dental care utilization between developed and developing countries.

In the present study, there was a significant positive association between age and DMFT (*p* < 0.001), which is comparable to another study done on pregnant women in Iran [21], and a study from India, where decayed teeth were influenced by age [28].

In this study more than half of the participants (64.1%) reported that they brushed their teeth once a day or more, which was far less than the pregnant women daily brushing frequency in Finland (90%), Australia (91%), Kuwait (92%), and England (73.7%) [27, 29–31]. Brushing habit in pregnancy is affected by nausea during pregnancy which may lead to decreased frequency of habit in this stage. However, it is very crucial because pregnant women who do not care enough to brush their own teeth will probably neglect cleaning their baby’s mouth [32, 33].

We found that higher education was associated with less dental caries. Our result confirmed the findings from previous studies from Iran [21, 34] and other countries [35–37] in which women with low level of education were more likely to have untreated dental caries compared to women with high level of education.

In our study, 67.3% of pregnant women consumed sweets once a day. A study on a group of Asian women living in England showed that about 63% of pregnant women during pregnancy increased consumption of sugar [38]. Women who consumed sweets more than once a day had significantly less dental fillings. This could be explained by possible clustering of dental care behaviors like more sweet consumption and less frequent dental visits documented elsewhere [12]. This finding is in accordance with previous evidence suggesting more number of filling in older expectant mothers who consume more sweets [38, 39]. Both sweet consumption and dental visit behaviors are usually affected by pregnancy. Pregnant women tend to use more sweets and avoid dental visit.

It was observed here that half of the studied pregnant women did not visit a dentist in the previous year, and those without dental visit had significantly less dental missing. This finding could be because of the role of dentist in teeth extraction; people should visit a dentist to remove their teeth. Similar to our findings, the American study reported that 35% of pregnant women who did not have a dental visit within the past year and 56% of women did not visit a dentist during pregnancy [40]. Studies reported that pregnant women experienced oral health problems and they did not often utilize dental care services during pregnancy [41, 42]. Many pregnant women believe that dental procedures are harmful for them and their fetuses [43]. In spite of highly subsidized dental care services for pregnant women in Iran public health care centers, pregnant women had less dental visits. It indicates the need for changing the attitude of expectant mothers in this regard.

BOP is an indicator of poor oral hygiene, and is of special importance in pregnancy, when inflammatory response to gingival bacteria is elevated [44–46]. Both BOP and periodontal pockets have been shown to affect pregnancy outcomes including increasing the chance of low birth weight and preterm birth [46].

This study was one of the few epidemiological studies on pregnant women in Iran. It benefited from good sample size from all health care centers of Pishva and Pakdasht including. The results of this study cannot be generalized to all Iranian pregnant women; however, it covers a representative sample in Varamin region.

However, some limitations should be considered in the current study. Some pregnant women did not show up for examinations although they were contacted up to three times. The researchers tried to encourage women for participation by providing free toothbrush and toothpastes. There were some oral health behaviors like using mouth rinse which were not studied in this research. We could not find any published evidence regarding the prevalence of mouth rinse use in Iran which informal references report it to be very low in Iranian population.

The other limitation of study was the cross-sectional design which did not allow us to study the chronological order of the risk factors and outcomes, not permitting study the causation, effect.

This study provides a snapshot of oral health status of pregnant women in a partially deprived area in Iran. The findings showed that a majority of expectant mothers had gingival inflammation, having a mean of seven decayed teeth in their mouth. This situation indicates high level of unfavorable bacteria in mothers’ mouth which will be transferrable to the newborns’ mouth in the future. The authors suggestions regarding these study findings are: 1- before pregnancy, women should be clinically examined and advised to get necessary dental treatments. It could be emphasized by midwives and gynecologists who are involved in maternity health. 2- during pregnancy, women’s oral hygiene education should be integrated in the common maternal care. It could be also of important step for the mothers to take the responsibility of oral health of their newborns.

## Conclusion

Oral health status of pregnant women was not satisfactory, having an average of seven decayed teeth in their mouth. Older women had less dental caries but apparently more missing teeth indicating improper received dental care.

## Data Availability

The data that support the findings of this study are available from the corresponding author upon reasonable request.

## Abbreviations

BOP: Bleeding On Probing
CI: Confidence Intervals
CR: Count Ratios
DMFT: Decayed, Missed, Filled Teeth
SD: Standard Deviation
WHO: World Health Organization
